# Evaluation of Idiopathic Choroidal Neovascularization with Indocyanine Green Angiography in Patients Undergoing Bevacizumab Therapy

**DOI:** 10.1155/2015/642624

**Published:** 2015-05-31

**Authors:** Ryan B. Rush, Sloan W. Rush

**Affiliations:** ^1^Southwest Retina Specialists, 7411 Wallace Boulevard, Amarillo, TX 79106, USA; ^2^Panhandle Eye Group, 7400 Fleming Avenue, Amarillo, TX 79106, USA; ^3^Texas Tech University Health Science Center, 1400 S. Coulter, Amarillo, TX 79106, USA

## Abstract

*Purpose*. To examine the clinical implications of change in choroidal neovascularization (CNV) size on indocyanine green (ICG) angiography in subjects with idiopathic CNV undergoing bevacizumab therapy.* Methods*. The charts of subjects with an idiopathic CNV treated by a modified PRN regimen with intravitreal bevacizumab over a 12-month period were retrospectively reviewed.* Results*. There were 34 subjects included in the analysis. Baseline CNV sizes of less than 1.0 mm^2^ on ICG angiography correlated with complete CNV resolution (*P* = 0.0404), fewer injections delivered (*P* = 0.0002), and better Snellen visual acuity (*P* = 0.0098) at 12 months. Subjects that experienced a 33% or more reduction in CNV size on ICG angiography at 2 months had complete CNV resolution (*P* = 0.0047) and fewer injections (*P* < 0.0001) at 12 months compared to subjects that did not experience a 33% or more reduction in CNV size on ICG angiography at 2 months.* Conclusions*. Smaller baseline CNV size on ICG angiography resulted in better visual acuity and fewer injections at 12 months, and a reduction of 33% or more in CNV size after 2 months may predict a better clinical course in subjects with idiopathic CNV undergoing bevacizumab therapy.

## 1. Introduction

Idiopathic choroidal neovascularization (CNV) is a diagnosis of exclusion occurring in subjects younger than 50 years without any observable ocular or systemic disease associations [1, 2]. Although permanent visual loss can occur without treatment, the natural history and visual outcomes of idiopathic CNV are generally more favorable than CNV attributable to age-related macular degeneration (AMD) [3]. Several studies have reported favorable visual and anatomic outcomes in subjects with idiopathic CNV undergoing antivascular endothelial growth factor (VEGF) therapy [4–10].

Baseline CNV size has been described as an important prognostic indicator of response to anti-VEGF therapy in neovascular AMD and pathologic myopia [11, 12]. Recently, indocyanine green (ICG) angiography with the Heidelberg Spectralis (Heidelberg Engineering, Heidelberg, Germany) system has been reported to be an accurate and reproducible method for obtaining measurements of CNV secondary to AMD and pathologic myopia [13, 14]. It remains unknown if change in CNV size in response to anti-VEGF therapy in patients with idiopathic CNV can predict the clinical course for these patients. In this study, the authors investigate the clinical correlations and implications that change in CNV size on ICG angiography has on treatment-naïve subjects with idiopathic CNV undergoing bevacizumab therapy during a 12-month follow-up interval.

## 2. Methods

The SRS institutional review board (IORG0007600/IRB00009122) approved this retrospective chart review of subjects with idiopathic CNV treated with bevacizumab from January 2009 to December 2014 at a single private-practice institution. All research components adhered to the tenets of the Declaration of Helsinki and were conducted in agreement with human research standards and regulations.

The inclusion criteria for the study were as follows: (1) age was <50 years; (2) a diagnosis of treatment-naïve subfoveal CNV was demonstrated by clinical examination, fluorescein angiography (FA), optical coherence tomography (OCT), and ICG angiography; (3) baseline Snellen best-corrected visual acuity (BCVA) was between 20/25 and 20/200; (4) baseline central macular thickness on OCT was >320 microns; (5) a 12-month follow-up period was fully documented with interval BCVA, examination details, OCT, FA, and ICG angiography; ICG angiography must have been performed at baseline, 2 months (± one month), 6 months (±2 months), and 12 months (±2 months); (6) a pro re nata (PRN) regimen was adhered to as described below; and (7) there were no identifiable primary ocular or systemic diseases associated with the CNV.

The exclusion criteria for the study were as follows: (1) photodynamic therapy, macular photocoagulation, or intravitreal injections other than bevacizumab to the study eye were given during the study period, (2) media opacity was significantly affecting BCVA or hindering retinal examination or imaging acquisition, (3) intraocular surgery for any indication was performed during the study period or within 3 months prior to subject enrollment, (4) visually significant retinal diseases other than idiopathic CNV, active uveitis, or uncontrolled glaucoma were present at enrollment or developed during the study period, and (5) a previous vitrectomy was performed at any time to the study eye (before or during the study period).

Baseline examinations included BCVA, an anterior and posterior segment slit-lamp examination, OCT, FA, and ICG angiography. Subjects received the initial bevacizumab injection during the baseline evaluation, and further bevacizumab injections were determined according to the following PRN schedule: consecutive monthly bevacizumab injections were given until the macula was dry (without intraretinal or subretinal fluid) on OCT and without macular hemorrhage on fundus examination. Once these measures were achieved, bevacizumab therapy would be deferred and the patient would return for follow-up four weeks later. If retinal fluid recurred on OCT, macular hemorrhage developed, or BCVA dropped by 2 or more lines after injection deferral, treatment would then be reinitiated. If subjects demonstrated resolution of CNV on ICG angiography or were without retinal edema on OCT for three consecutive months, follow-up was extended out to 8 (±4) weeks' interval.

The Heidelberg Spectralis system was used for all OCT, FA, and ICG angiography procedures. Intraretinal and/or subretinal fluid and central macular thickness were determined by OCT. Central macular thickness was obtained from the center subfield of the macular thickness map. The technique for determining CNV size on ICG angiography with the Heidelberg Spectralis surface area-measuring software has been previously described [14]. Briefly, an early to midframe (within the first three minutes) ICG angiography test image was selected for analysis. The visible CNV margins were manually encircled by two masked observers independently using the “Inlay” function, followed by surface area computation with the software. If CNV measurements did not agree within 10% of the lesion size between masked observers, the patient was then excluded from the analysis. CNV was considered resolved when the CNV could no longer be visualized on ICG angiography by either masked observer.

### 2.1. Main Outcomes and Statistical Analysis

The study's main outcomes were CNV surface area change on ICG angiography at 2 months, 6 months, and 12 months. The secondary outcomes were the correlation of change in CNV surface area on ICG angiography with change in BCVA, change in central macular thickness on OCT, and the number of injections delivered over the 12-month study interval. Response to treatment was considered significant when the CNV surface area change on ICG angiography was 33% or more from the selected follow-up interval. The JMP software package (JMP Version 10, SAS Institute, Cary, NC, USA) was used for statistical calculations. Snellen BCVA was changed into a logarithm of the minimal angle of resolution (logMAR). Nominal variables were compared by Chi-Square and Likelihood Ratios, and numerical means were compared by one-way analysis of variance (ANOVA). A probability of <0.05 was deemed statistically significant.

## 3. Results

There were 37 subjects that met the study's inclusion/exclusion criteria. Of these 37 subjects, 34 were included in the data analysis after CNV measurements were assessed for interobserver agreement, thereby resulting in 91.8% (34/37) concordance between masked observers within 10% of the lesion size on ICG angiography. As a comparative check on ICG angiography, interobserver agreement within 10% of the lesion size on FA was 67.5% (25/37). The means and distributions of the study population's baseline characteristics are summarized in Table 1. Baseline CNV size on FA was overestimated by 22.7% (2.36 mm^2^ versus 1.82 mm^2^) when compared to ICG angiography.

There was a mean of 3.7 (±1.9) injections delivered over the 12-month study interval. The mean BCVA at 12 months was 0.34 (±0.24) logMAR (20/44 Snellen), and the mean central macular thickness on OCT at 12 months was 278.3 (±36.1) microns. The changes in BCVA and central macular thickness on OCT during the study interval were significantly improved (*P* = 0.0072 and *P* < 0.0001, resp.). A total of 26.4% of subjects (9/34) improved 3 or more lines of BCVA at 12 months. There were no subjects that experienced a loss of 3 or more lines of BCVA during the study interval.

**Figure 1:**
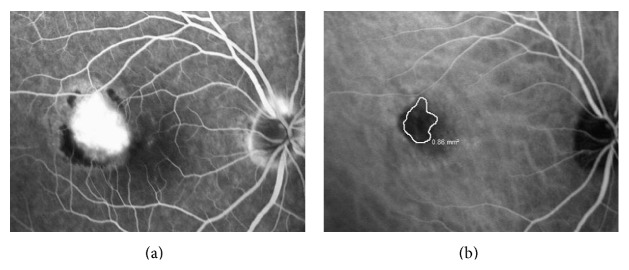
The baseline fluorescein angiography (a) and indocyanine green angiography (b) images of a 37-year-old subject with subfoveal idiopathic choroidal neovascularization. The choroidal neovascularization is encircled in white with the corresponding surface area calculation on the indocyanine green angiography image. The Snellen visual acuity was 20/80.

**Figure 2:**
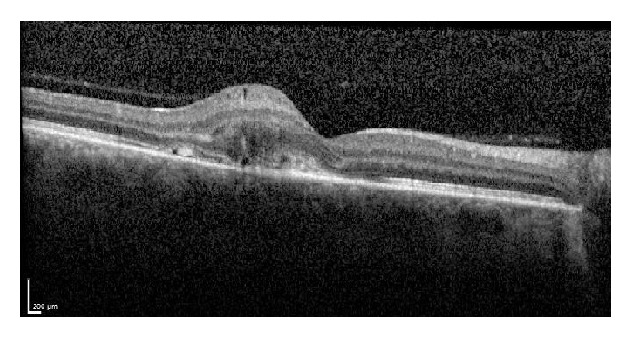
The baseline optical coherence tomography image of the same subject from Figure 1. Notice the presence of retinal fluid and choroidal neovascularization in the subretinal space.

**Figure 3:**
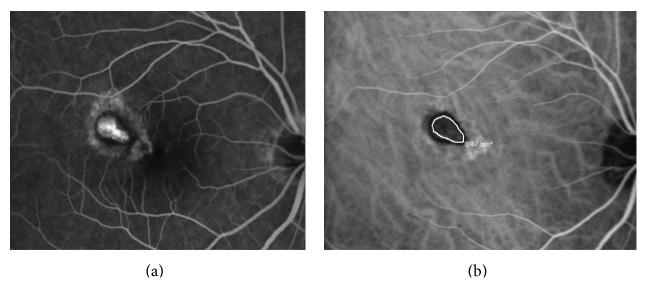
The fluorescein angiography (a) and indocyanine green angiography (b) images of the same subject presented in Figures 1 and 2 two months after two consecutive monthly bevacizumab injections were given. The choroidal neovascularization has decreased 45.3% ((0.86 − 0.47)/0.86) from its baseline value on indocyanine green angiography. The Snellen visual acuity improved to 20/30.

**Figure 4:**
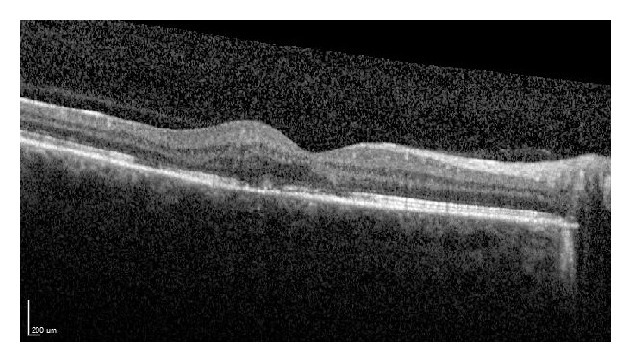
The optical coherence tomography image of the same subject presented in Figures 1–3 two months after two consecutive monthly bevacizumab injections were given. Notice the resolution of most of the retinal fluid and the choroidal neovascularization diminishment within the subretinal space.

The mean CNV surface areas on ICG angiography were 1.11 (±1.58), 0.33 (±0.58), and 0.15 (±0.42) mm^2^ at 2 months, 6 months, and 12 months, respectively. The change in CNV size on ICG angiography from baseline was significantly improved at 6 months and 12 months (*P* < 0.0001 for both), but not at 2 months (*P* = 0.1207). A total of 35.2% (12/34) of subjects had complete CNV resolution on ICG angiography at 2 months, 70.5% (24/34) at 6 months, and 85.2% (29/34) at 12 months. A total of 11.7% (4/34) of subjects only required one injection to achieve complete CNV resolution on ICG angiography during the 12-month study interval, and a total of 26.4% (9/34) of subjects required two or fewer injections to achieve complete CNV resolution on ICG angiography during the 12-month study interval. The recurrence rate of CNV on ICG angiography after 3 monthly bevacizumab injections was 32.3% (11/34) during the study interval. A total of 61.7% (21/34) of subjects had a 33% or more CNV size reduction on ICG angiography at 2 months; subjects that experienced a 33% or more reduction in CNV size on ICG angiography at 2 months had more complete CNV resolution at 12 months (*P* = 0.0047) and fewer injections delivered during the study interval (*P* < 0.0001), but not better visual acuity (*P* = 0.0721) or a lower central macular thickness on OCT (*P* = 0.7656) at the end of the 12-month study interval compared to those subjects without a 33% or more reduction at 2 months. Using linear regression analysis, change in CNV size on ICG angiography correlated poorly with change in BCVA (*P* = 0.6836; *R* ^2^ = 0.006) but correlated well with change in central macular thickness on OCT (*P* = 0.0452; *R* ^2^ = 0.14) during the study interval. See Figures 1–4 for a case example.

Baseline CNV sizes of less than 1.0 mm^2^ on ICG angiography significantly correlated with complete CNV resolution at 12 months (*P* = 0.0404), fewer injections delivered during the study interval (*P* = 0.0002), and better visual acuity (*P* = 0.0098), but not lower central macular thickness on OCT (*P* = 0.2899) at the end of the 12-month study interval. Overall, 100% of subjects that had a baseline CNV size on ICG angiography of less than 1.0 mm^2^ had complete CNV resolution at 12 months.

## 4. Discussion

The baseline characteristics and overall outcomes in our study were broadly on par to other idiopathic CNV studies evaluating response to anti-VEGF therapy [4–10]. Similar to the mean 3.3 injections delivered over the first 12 months by Kang and Koh [4], our study subjects received 3.7 injections during the 12-month study interval. Our study is one of the largest case series of idiopathic CNV subjects undergoing anti-VEGF therapy, and the favorable visual and anatomic outcomes achieved in our study lend further confidence in the effectiveness of intravitreal bevacizumab for the management of idiopathic CNV.

This is the first study to investigate change in CNV size on ICG angiography in treatment-naïve patients with idiopathic CNV undergoing anti-VEGF therapy; previous studies either did not report CNV size [5–8] or measured CNV size with FA, reporting only the greatest linear dimension of the CNV leakage [4]. The authors of this study assessed CNV surface area on ICG angiography with the Heidelberg Spectralis software, believing this would provide a more objective and reproducible method for determining CNV size compared to a FA classification system based on determination of hyperfluorescence and dye leakage. Indeed, when FA was used to measure CNV surface area during our study as a comparative check on ICG angiography, masked interobserver agreement was notably less and CNV size was evidently overestimated. ICG angiography has been reported to improve the detectability of CNV by 55–61% compared to FA in neovascular AMD patients, especially when occult CNV was present [15–19]. The authors believe that the measurement discrepancy between ICG angiography and FA can be best explained by noting that ICG angiography allows a direct measurement of the actual CNV lesion, whereas FA only allows an indirect measurement of the secondary effects of CNV such as retinal leaking and staining.

A reduction in CNV size on ICG angiography of 33% or more at 2-month follow-up predicted a favorable clinical course with fewer injections needed and more CNV resolution in the study population by the end of the 12-month study interval. Change in CNV size during the study interval correlated well with change in central macular thickness and total injection number, and subjects with smaller baseline CNV surface areas were more likely to have favorable visual outcomes with fewer injections; this highlights the importance of early detection and treatment while the CNV size remains small in order to get the best final outcomes. Change in CNV size did not correlate well with change in visual acuity, and this is most likely because patients with larger baseline CNV and increased baseline central macular thickness stand to gain the most in net anatomic improvement with treatment, while having the worst potential for visual recovery on account of their disease being more advanced prior to treatment.

The mean baseline CNV size in our study population with idiopathic CNV was more analogous to that reported in myopic CNV rather than neovascular AMD [11–14]. Baseline CNV surface area measured on ICG angiography has ranged from 0.93 to 2.18 mm^2^ in myopic subjects [20–22] and from 1.9 to 7.1 mm^2^ in neovascular AMD subjects [14, 23]. Change in CNV size in our study patients in response to anti-VEGF therapy was also more comparable to what has been reported in myopic CNV subjects undergoing anti-VEGF therapy [13, 20–22]. Similar to Yang et al. [13], greater baseline CNV size in our study subjects correlated with the need for a higher number of bevacizumab injections during the study interval. The recurrence rate of CNV on ICG angiography after 3 monthly bevacizumab injections in our study was similar to the rates reported by Yang et al. [13] (23.3%) and Lai et al. [20] (22.7%) in eyes with myopic CNV after 3 monthly anti-VEGF injections. However, complete CNV resolution after just 1-2 monthly anti-VEGF injections was lower in our study compared to the resolution rate of 54.7% reported by Hayashi et al. [21] in subjects with myopic CNV. Nevertheless, complete CNV resolution on ICG angiography in subjects with neovascular AMD has been reported to be only 7.3% at 12-month follow-up [14]. Therefore according to baseline CNV size and change in CNV size in response to anti-VEGF therapy, idiopathic CNV appears to behave clinically more like myopic CNV rather than neovascular AMD.

Weaknesses of this study include the use of logMAR visual acuity, the retrospective collection of data, and the observer-dependent CNV surface area determination on ICG angiography. In conclusion, our study demonstrates that smaller baseline CNV surface area on ICG angiography results in better visual acuity and fewer injections at 12 months in subjects with idiopathic CNV and that a reduction of 33% or more in CNV size after 2-month follow-up predicts a better clinical course with fewer injections at 12 months. Future prospective studies are needed to further validate these findings.

## Figures and Tables

**Table 1:** The baseline means and distributions of the study population.

Variable	Outcome
Age (years)	38.7 (±10.0)

Gender	Female: 19 (55.8%)
	Male: 15 (44.1%)

Best-corrected visual acuity (logMAR)	0.52 (±0.20)

Central macular thickness on optical coherence tomography (microns)	355.8 (±38.3)

Choroidal neovascularization surface on indocyanine green angiography (mm^2^)	1.82 (±1.69)

Choroidal neovascularization surface on fluorescein angiography (mm^2^)	2.36 (±1.91)
